# Association of *SIX1/SIX6* locus polymorphisms with regional circumpapillary retinal nerve fibre layer thickness: The Nagahama study

**DOI:** 10.1038/s41598-017-02299-7

**Published:** 2017-06-29

**Authors:** Munemitsu Yoshikawa, Kenji Yamashiro, Hideo Nakanishi, Manabu Miyata, Masahiro Miyake, Yoshikatsu Hosoda, Yasuharu Tabara, Fumihiko Matsuda, Nagahisa Yoshimura, the Nagahama Study Group, Takahisa Kawaguchi, Kazuya Setoh, Yoshimitsu Takahashi, Shinji Kosugi, Takeo Nakayama

**Affiliations:** 1 Department of Ophthalmology and Visual Sciences, Kyoto University Graduate School of Medicine, 54 Kawahara, Shogoin, Sakyo, Kyoto, 606-8507 Japan; 2 Department of Ophthalmology, Otsu Red Cross Hospital, 1-1-35 Nagara, Otsu, 520-8511 Japan; 3 Center for Genomic Medicine, Kyoto University Graduate School of Medicine, 54 Kawahara, Shogoin, Sakyo, Kyoto 606-8507 Japan; 4 Department of Health Informatics, Kyoto University Graduate School of Medicine, 54 Kawahara, Shogoin, Sakyo, Kyoto 606-8507 Japan; 5 Department of Medical Ethics and Medical Genetics, Kyoto University Graduate School of Medicine, 54 Kawahara, Shogoin, Sakyo, Kyoto 606-8507 Japan

## Abstract

*SIX1 and SIX6* are glaucoma susceptibility genes. Previous reports indicate that the single nucleotide polymorphism (SNP) rs33912345 in *SIX6* is associated with inferior circumpapillary retinal nerve fibre layer (cpRNFL) thickness (cpRNFLT). Although the region of visual field defect in glaucoma patients is directly related to cpRNFL thinning, a detailed sector analysis has not been performed in genetic association studies. In the present study, we evaluated 26 tagging SNPs in the *SIX1/SIX6* locus ±50 kb region in a population of 2,306 Japanese subjects with 4- and 32-sector cpRNFLT analysis. While no SNPs showed a significant association with cpRNFLT in the 4-sectored analysis, the finer 32-sector assessment clearly showed a significant association between rs33912345 in the *SIX1/SIX6* locus with inferior cpRNFL thinning at 292.5–303.8° (β = −4.55, *P* = 3.0 × 10^−5^). Furthermore, the fine-sectored cpRNFLT analysis indicated that *SIX1/SIX6* polymorphisms would affect cpRNFL thinning at 281.3–303.8°, which corresponds to parafoveal scotoma in a visual field test of glaucoma patients.

## Introduction

Glaucoma is a complex vision-threatening disorder with a multifactorial aetiology that includes both genetic and environmental factors^1–3^, and it is one of the most prevalent causes of irreversible blindness worldwide^4^. To date, various glaucoma susceptibility genes have been identified by genome-wide association studies (GWAS)^5–13^. While the majority of associated genes have been characterized^5–8^, GWAS for glaucoma endophenotypes—such as vertical cup-to-disc ratio (VCDR)^9, 10^ and intraocular pressure (IOP)^11–13^—have also contributed to the discovery of glaucoma susceptibility genes, indicative of endophenotype-specific genetic associations. Of the known glaucoma susceptibility genes, several have been shown to exhibit associations with race^14^, sex^15, 16^, and the location of the visual field defect (VFD)^17–20^.

Although glaucomatous VFD generally occurs in the upper hemifield, early involvement of the inferior hemifield and/or the paracentral VFD has been observed in certain subgroups with low-tension glaucoma or myopia, resulting in severely impaired quality of vision (QOV) from the initial stages of disease^21–25^. Therefore, the ability to predict the risk of these QOV-threatening VFD patterns using specific genetic associations to the locations of the VFD would be highly beneficial. However, current visual field testing is based on subjective patient responses and can be susceptible to various extraneous factors, such as cataract, dementia, concentration, and physical conditions, thus preventing an accurate evaluation of the associations between susceptibility genes and VFD patterns.

Recent advances in optical coherence tomography (OCT) have allowed ophthalmologists to perform quantitative evaluations of circumpapillary retinal nerve fibre layer thickness (cpRNFLT), which represents glaucomatous optic neuropathy (GON) with high reproducibility and reliability. This morphological testing method generates objective data less influenced by the problems listed above^26^. The association between cpRNFLT and VFD pattern is highly correlative^27–29^, suggesting that cpRNFLT would be a better measure to evaluate the genetic contribution to GON endophenotype than visual field testing.

To date, genetic studies on cpRNFLT have shown consistent contributions of the single nucleotide polymorphisms (SNPs) rs33912345 and rs10483727 located within the *SIX1/SIX6* loci to cpRNFL thinning in the superior and inferior sectors, but not in the temporal, nasal, and global sectors^30–32^. The significance of the *SIX1/SIX6* locus in glaucoma was initially discovered by GWAS for VCDR and primary open-angle glaucoma (POAG), and subsequent studies confirmed the association of polymorphisms in this region with glaucoma onset.^9, 33, 34^ According to previous GON structure-function correlations^27–29^, a finer cpRNFLT analysis with >4 sectors would be better suited to fully evaluate the risk of QOV-threatening VFD patterns. Therefore, the present study examined genetic associations of the *SIX1/SIX6* locus with cpRNFLT using 4- and 32-sector analyses.

## Results

### Study population

Participants were excluded due to prior intraocular surgery (N = 125), axial length ≥26 mm (N = 258), presence of other ocular disease (N = 64), and cpRNFL image quality (N = 54) as described in the Methods section. A total of 2,306 subjects passed the exclusion criteria and the participant demographics are shown in Table 1. The average age was 57.6 ± 13.6 years (mean ± SD; range, 34–80 years). Mean axial length of the right eye was 23.78 ± 1.04 mm (range, 16.46–25.99 mm). Two-thirds of the participants (68.9%) were females; however, this did not affect global cpRNFLT after the adjustment for age and axial length. The mean global cpRNFLT was 101.6 ± 12.0 μm, and 122.4 ± 20.0 μm, 132.1 ± 20.8 μm, 76.3 ± 13.9 μm, and 75.5 ± 14.3 μm in the superior, inferior, temporal, and nasal sectors, respectively.

**Table 1:** Demographics of study participants.

Number	2,306
Age (years)	57.6 ± 13.6
Sex (male/female)	717/1589
IOP (mmHg)^†^	14.55 ± 3.00
Axial length (mm)^†^	23.78 ± 1.04
Central corneal thickness (μm)^†^	543.3 ± 28.6
cpRNFLT (μm)^†^	
*Global*	101.6 ± 12.0
*Temporal*	76.3 ± 13.9
*Superior*	122.4 ± 20.0
*Nasal*	75.5 ± 14.3
*Inferior*	132.1 ± 20.8

Data represent the mean ± SD. IOP, intraocular pressure, cpRNFLT, circumpapillary retinal nerve fibre layer thickness. ^†^Data were acquired from the right eye.

### Association of SIX1/SIX6 polymorphisms with regional cpRNFLT

Association of the 26 tagging SNPs along the 4 cpRNFLT sectors are shown in Table 2. Only rs12147345 showed a marginal association with cpRNFLT in the temporal region (β = 1.08; 95% confidential interval [CI], 0.26–1.90; *P = *0.010). On the other hand, a finer assessment with 32 sectors found 5 additional *SIX1/SIX6* SNPs, including rs33912345, to be significantly or marginally associated with a cpRNFLT region, mostly in inferior sectors (Table 3). Among these 6 SNPs, only rs148908311 showed a marginal association with cpRNFLT in the superior region, whereas the other 5 SNPs showed significant or marginal associations with cpRNFLT in the inferior regions. The strongest and only significant association (P < 6.0 × 10^−5^; 0.05/26 SNPs/32 sectors) was observed between rs33912345 and cpRNFLT in the inferior region at 292.5–303.8° (β = −4.55; 95% CI, −2.42–−6.69), *P* = 3.0 × 10^−5^). For all SNPs that were significantly or marginally associated with regional cpRNFLTs, we confirmed identical effect directions of the genetic variances to the regional cpRNFLT as to the global cpRNFLT.

**Table 2:** Associations of a *SIX1/SIX6* locus and circumpapillary retinal nerve fibre layer thickness including 4 sectors.

CHR	SNP	BP*	Gene	Minor Allele*	Other Allele*	Global		Temporal		Superior		Nasal		Inferior	
						Beta	P†	Beta	P†	Beta	P†	Beta	P†	Beta	P†
14	rs140399719_T_C	60940196	*SIX1/SIX6*	T	C	−0.76	0.30	−0.69	0.42	1.48	0.22	−1.40	0.11	−2.42	0.056
14	rs144039268_G_T	60940392	*SIX1/SIX6*	G	T	−0.09	0.92	0.63	0.50	0.62	0.65	−0.75	0.45	−0.85	0.55
14	rs148908311_T_A	60941172	*SIX1/SIX6*	T	A	0.24	0.80	−1.02	0.35	2.43	0.12	0.24	0.84	−0.68	0.68
14	rs10138913_C_T	60943106	*SIX1/SIX6*	C	T	−0.46	0.18	−0.72	0.065	−0.09	0.87	0.29	0.48	−1.31	0.027
14	rs33912345_A_C	60976537	*SIX1/SIX6*	A	C	−0.52	0.18	−1.11	0.013	−0.23	0.71	0.67	0.15	−1.41	0.035
14	rs76172201_T_A	60989902	*SIX1/SIX6*	T	A	−0.38	0.65	0.93	0.34	−0.08	0.95	−1.78	0.08	−0.60	0.68
14	rs17097602_T_C	60997130	*SIX1/SIX6*	T	C	0.40	0.23	0.45	0.24	0.21	0.70	−0.11	0.79	1.03	0.073
14	rs1955691_G_A	61004128	*SIX1/SIX6*	G	A	0.03	0.95	0.37	0.47	−0.27	0.71	−0.32	0.55	0.34	0.66
14	rs1010053_A_G	61005625	*SIX1/SIX6*	A	G	−0.33	0.31	−0.50	0.18	−0.19	0.73	0.41	0.30	−1.06	0.061
14	rs12587483_A_G	61021885	*SIX1/SIX6*	A	G	0.38	0.26	0.35	0.37	0.08	0.88	−0.26	0.53	1.34	0.022
14	rs10133871_A_C	61063771	*SIX1/SIX6*	A	C	0.16	0.67	0.52	0.24	0.21	0.74	−0.06	0.90	−0.02	0.98
14	rs77636526_A_G	61077496	*SIX1/SIX6*	A	G	−2.25	0.046	−2.59	0.046	−0.88	0.63	−2.85	0.036	−2.67	0.17
14	rs79319089_A_T	61080521	*SIX1/SIX6*	A	T	−0.52	0.50	−0.65	0.47	0.20	0.88	−0.20	0.83	−1.43	0.28
14	rs75320987_C_T	61085911	*SIX1/SIX6*	C	T	0.82	0.15	0.70	0.28	−0.02	0.98	1.06	0.12	1.52	0.12
14	rs2057136_T_A	61106019	*SIX1/SIX6*	T	A	0.29	0.48	0.31	0.52	0.09	0.90	−0.12	0.80	0.90	0.21
14	rs117183588_T_A	61120441	*SIX1/SIX6*	T	A	−0.74	0.35	−1.02	0.26	0.62	0.64	−1.50	0.12	−1.06	0.44
14	rs7153648_C_G	61122526	*SIX1/SIX6*	C	G	0.20	0.61	0.44	0.32	0.14	0.83	−0.43	0.36	0.65	0.34
14	rs73309474_C_A	61124161	*SIX1/SIX6*	C	A	−1.06	0.21	−1.76	0.070	−0.11	0.94	−1.15	0.26	−1.20	0.41
14	rs143331462_T_C	61124545	*SIX1/SIX6*	T	C	0.03	0.96	0.99	0.22	0.80	0.49	−0.82	0.33	−0.83	0.50
14	rs7143029_T_C	61127916	*SIX1/SIX6*	T	C	0.45	0.22	1.04	0.013	0.34	0.57	−0.28	0.52	0.71	0.26
14	rs12147345_C_T	61140406	*SIX1/SIX6*	C	T	0.48	0.19	**1.08**	**0.010**	0.42	0.49	−0.30	0.50	0.73	0.25
14	rs12147346_C_T	61140408	*SIX1/SIX6*	C	T	0.39	0.27	0.81	0.047	0.42	0.47	−0.32	0.46	0.66	0.28
14	rs10137383_T_A	61140517	*SIX1/SIX6*	T	A	−0.29	0.54	−0.81	0.13	−0.06	0.94	−0.09	0.88	−0.21	0.80
14	rs12589826_A_G	61143058	*SIX1/SIX6*	A	G	0.42	0.23	0.88	0.031	0.49	0.40	−0.31	0.46	0.64	0.29
14	rs2351179_C_T	61157015	*SIX1/SIX6*	C	T	−0.25	0.44	0.05	0.90	0.11	0.83	−0.48	0.23	−0.70	0.22
14	rs61991690_C_T	61160190	*SIX1/SIX6*	C	T	0.42	0.36	0.27	0.60	0.29	0.70	0.20	0.72	0.91	0.25

CHR, chromosome; SNP, single nucleotide polymorphism; BP, base pair.

*Positions and alleles are given relative to the positive strand of NCBI build 37 of the human genome.

^†^Linear regression analyses were applied assuming additive effect of the per minor allele variant, adjusted for age and sex. Significant (P < 4.8 × 10^−4^) or suggestive (P < 0.0125) associations are shown in bold.

**Table 3:** Associations of *SIX1/SIX6* locus polymorphisms and circumpapillary retinal nerve fibre layer thickness in 32 sectors.

SNP	RNFL 01	RNFL 02	RNFL 03	RNFL 04	RNFL 05	RNFL 06	RNFL 07	RNFL 08	RNFL 09	RNFL 10	RNFL 11	RNFL 12	RNFL 13	RNFL 14	RNFL 15	RNFL 16
	P*	P*	P*	P*	P*	P*	P*	P*	P*	P*	P*	P*	P*	P*	P*	P*
rs140399719	0.11	0.33	0.14	0.018	0.074	0.51	0.24	0.038	0.016	0.026	0.12	0.80	0.17	0.25	0.33	0.99
rs144039268	0.99	0.87	0.79	0.54	0.33	0.17	0.37	0.62	0.64	0.79	0.26	0.12	0.12	0.29	0.63	0.71
rs148908311	0.31	0.10	0.13	0.22	0.29	0.64	0.050	**0.0011**	0.022	0.57	0.81	0.29	0.17	0.22	0.68	0.72
rs10138913	0.12	0.060	0.15	0.21	0.45	0.21	0.79	0.058	0.057	0.76	0.12	0.19	0.34	0.68	0.096	0.012
rs33912345	0.14	0.067	0.08	0.054	0.089	0.026	0.57	0.050	0.039	0.54	0.25	0.84	0.70	0.087	0.011	0.0030
rs76172201	0.63	0.46	0.51	0.55	0.30	0.34	0.83	0.46	0.23	0.93	0.94	0.44	0.20	0.23	0.075	0.10
rs17097602	0.19	0.26	0.62	0.80	0.81	0.42	0.43	0.51	0.50	0.80	0.17	0.44	0.60	0.70	0.34	0.14
rs1955691	0.77	0.33	0.25	0.20	0.18	0.54	0.48	0.077	0.08	0.99	0.88	0.53	0.67	0.88	0.31	0.16
rs1010053	0.38	0.57	0.81	0.65	0.74	0.23	0.49	0.69	0.69	0.60	0.56	0.51	0.26	0.072	0.078	0.18
rs12587483	0.27	0.42	0.97	0.91	0.62	0.58	0.60	0.52	0.59	0.73	0.27	0.74	0.96	0.37	0.19	0.16
rs10133871	0.54	0.17	0.084	0.081	0.10	0.35	0.97	0.22	0.16	0.83	0.43	0.18	0.26	0.62	0.68	0.18
rs77636526	0.02	0.064	0.20	0.67	0.88	0.98	0.28	0.78	0.017	0.19	0.98	0.74	0.44	0.07	0.016	0.018
rs79319089	0.38	0.43	0.72	0.96	0.91	0.51	0.23	0.80	0.24	0.60	0.73	0.70	0.98	0.84	0.77	0.96
rs75320987	0.35	0.15	0.12	0.16	0.32	0.82	0.18	0.23	0.87	0.58	0.83	0.23	0.042	0.058	0.29	0.82
rs2057136	0.88	0.39	0.32	0.20	0.13	0.26	0.62	0.21	0.31	0.93	0.84	0.29	0.26	0.78	0.33	0.075
rs117183588	0.024	0.069	0.21	0.65	0.65	0.37	0.32	0.04	0.41	0.58	0.96	0.35	0.08	0.044	0.13	0.59
rs7153648	0.73	0.36	0.33	0.24	0.32	0.83	0.78	0.66	0.74	0.39	0.76	0.93	0.83	0.78	0.14	0.036
rs73309474	0.0098	0.04	0.07	0.18	0.31	0.17	0.58	0.11	0.86	0.89	0.98	0.59	0.28	0.13	0.16	0.45
rs143331462	0.96	0.81	0.35	0.16	0.15	0.31	0.49	0.52	0.38	0.64	0.29	0.34	0.38	0.69	0.71	0.63
rs7143029	0.087	0.036	0.020	0.030	0.044	0.11	0.76	0.58	0.80	0.56	0.68	0.73	0.86	0.87	0.50	0.16
rs12147345	0.070	0.029	0.018	0.023	0.034	0.11	0.75	0.67	0.88	0.48	0.77	0.79	0.86	0.89	0.45	0.14
rs12147346	0.34	0.11	0.070	0.073	0.082	0.27	0.67	0.85	0.94	0.44	0.80	0.67	0.78	0.96	0.39	0.18
rs10137383	0.21	0.14	0.085	0.20	0.19	0.33	0.56	0.55	0.88	0.74	0.48	0.59	0.62	0.81	0.52	0.98
rs12589826	0.28	0.090	0.055	0.056	0.074	0.27	0.60	0.73	0.81	0.36	0.84	0.68	0.79	0.98	0.39	0.18
rs2351179	0.50	0.97	0.96	0.76	0.82	0.95	0.94	0.55	0.34	0.50	0.45	0.42	0.44	0.60	0.28	0.20
rs61991690	0.44	0.80	0.90	0.70	0.54	0.76	0.60	0.98	0.75	0.63	0.13	0.63	0.86	0.80	0.42	0.64
**SNP**	**RNFL 17**	**RNFL 18**	**RNFL 19**	**RNFL 20**	**RNFL 21**	**RNFL 22**	**RNFL 23**	**RNFL 24**	**RNFL 25**	**RNFL 26**	**RNFL 27**	**RNFL 28**	**RNFL 29**	**RNFL 30**	**RNFL 31**	**RNFL 32**
	**P** *	**P** *	**P** *	**P** *	**P** *	**P** *	**P** *	**P** *	**P** *	**P** *	**P** *	**P** *	**P** *	**P** *	**P** *	**P** *
rs140399719	0.96	0.14	0.023	0.031	0.14	0.44	0.85	0.41	0.015	0.0031	0.18	0.99	0.38	0.12	0.89	0.13
rs144039268	0.82	0.58	0.65	0.67	0.45	0.64	0.64	0.26	0.23	0.56	0.68	0.33	0.26	0.28	0.41	0.74
rs148908311	0.82	0.60	0.24	0.21	0.39	0.99	0.49	0.31	0.94	0.28	0.20	0.48	0.80	0.95	0.98	0.66
rs10138913	0.025	0.37	0.86	0.47	0.54	0.94	0.70	0.60	0.018	**2.9 × 10** ^**−4**^	0.0035	0.14	0.10	0.088	0.081	0.20
rs33912345	0.019	0.64	0.55	0.79	0.77	0.43	0.23	0.61	0.07	**8.1 × 10** ^**−5**^	**3.0 × 10** ^**−5**^	0.0027	0.0027	0.012	0.031	0.14
rs76172201	0.10	0.20	0.17	0.29	0.44	0.70	0.72	0.78	0.65	0.73	0.99	0.81	0.52	0.27	0.15	0.22
rs17097602	0.47	0.98	0.77	0.63	0.50	0.85	0.91	0.46	0.034	0.0071	0.069	0.36	0.20	0.20	0.16	0.16
rs1955691	0.045	0.25	1.00	0.67	0.89	0.66	0.71	0.78	0.74	0.25	0.21	0.60	0.83	0.79	0.83	0.79
rs1010053	0.77	0.63	0.66	0.72	0.71	0.63	0.71	0.99	0.082	0.0016	0.0020	0.021	0.023	0.078	0.15	0.18
rs12587483	0.56	0.96	0.92	0.91	0.67	0.93	0.77	0.32	0.010	**5.0 × 10** ^**−4**^	0.014	0.26	0.24	0.27	0.23	0.17
rs10133871	0.05	0.32	0.88	0.50	0.96	0.77	0.46	0.67	0.86	0.70	0.68	0.80	0.64	0.55	0.59	0.98
rs77636526	0.12	0.18	0.16	0.10	0.02	0.070	0.41	0.70	0.87	0.93	0.34	0.048	0.043	0.038	0.023	0.029
rs79319089	0.99	0.69	0.52	0.36	0.24	0.45	0.31	0.82	0.92	0.33	0.28	0.56	0.79	0.37	0.19	0.21
rs75320987	0.37	0.94	0.084	0.03	0.18	0.62	0.82	0.85	0.38	0.047	0.044	0.27	0.58	0.61	0.73	0.87
rs2057136	0.016	0.18	0.65	0.21	0.40	0.86	1.00	0.72	0.20	0.036	0.14	0.79	0.86	0.71	0.84	0.66
rs117183588	0.41	0.18	0.36	0.62	0.27	0.49	0.78	0.85	0.58	0.34	0.46	0.48	0.74	0.96	0.51	0.074
rs7153648	0.0029	0.067	0.85	0.37	0.96	0.56	1.00	0.72	0.45	0.13	0.066	0.45	0.54	0.28	0.29	0.90
rs73309474	0.42	0.36	0.77	0.87	0.46	0.62	0.86	0.96	0.74	0.55	0.30	0.15	0.28	0.59	0.23	0.034
rs143331462	0.65	0.56	0.24	0.004	0.0084	0.04	0.029	0.061	0.67	0.27	0.062	0.13	0.069	0.13	0.38	0.80
rs7143029	0.070	0.51	0.96	0.80	0.31	0.23	0.34	0.61	0.45	0.025	**0.0013**	0.013	0.029	0.033	0.036	0.13
rs12147345	0.070	0.51	0.95	0.78	0.30	0.20	0.32	0.59	0.43	0.019	**9.1 × 10** ^**−4**^	0.010	0.024	0.028	0.030	0.11
rs12147346	0.072	0.42	0.92	0.79	0.30	0.29	0.56	0.75	0.51	0.033	0.0036	0.035	0.051	0.038	0.089	0.44
rs10137383	0.93	0.52	0.55	0.82	0.47	0.58	0.22	0.43	0.61	0.44	0.14	0.16	0.30	0.40	0.23	0.21
rs12589826	0.070	0.44	0.93	0.75	0.27	0.30	0.62	0.73	0.55	0.039	0.0042	0.032	0.036	0.022	0.057	0.36
rs2351179	0.094	0.37	0.61	0.26	0.051	0.10	0.17	0.18	0.49	0.51	0.99	0.55	0.37	0.28	0.74	0.32
rs61991690	0.41	0.90	0.76	0.95	0.49	0.50	0.77	0.80	0.53	0.16	0.21	0.34	0.36	0.42	0.35	0.28

SNP, single nucleotide polymorphism; RNFL, retinal nerve fibre layer.

RNFL01–32 starts from the temporal region at 0–11.25° and at 11.25° (=360°/32 sectors) interval (clockwise direction).

*Linear regression analyses were applied assuming additive effect of the per minor allele variant, adjusted for age and sex. Significant (P < 6.0 × 10^−5^) or suggestive (<1.6 × 10^−3^) associations are shown in bold.

We observed that rs10483727—the only *SIX1/SIX6* SNP previously associated with glaucoma susceptibility by GWAS—was in close linkage disequilibrium (LD) with rs33912345 (R-sq = 0.99 in the JPT 1000 genomes dataset). Although rs10483727 was not included in the tagging SNPs in the present study, the C risk allele of rs33912345 (corresponding to the T risk allele of rs10483727) resulted in both regional and global cpRNFL thinning.

## Discussion

Our study used a tagging SNP approach to show that polymorphisms in the *SIX1/SIX6* region was significantly associated with inferior cpRNFLT and marginally associated with superior cpRNFLT in a community-based Japanese cohort. These results were consistent with previous candidate SNP evaluations on rs33912345^30–32^. In addition, our findings suggest that the 32-sector region-based approach for cpRNFLT enables the detection of underpowered and undermined genetic associations in 4-sector analyses.

The importance of the *SIX1/SIX6* locus in glaucoma was initially discovered by a GWAS for VCDR, and subsequent GWAS for POAG confirmed the association between polymorphisms in this locus with glaucoma onset^9, 33, 34^. This is the only locus where an association with cpRNFLT has been established^30–32^. In this study, our detailed analysis further specified the cpRNFL region of association, and revealed that *SIX1/SIX6* affects cpRNFL thinning at 281.3–303.8° among the inferior region, which may have clinical and biological relevance since retinal nerve fibre layer defects also occur in this region. In addition, according to the Garway-Heath map^27^, RNFL thinning of this region would lead to upper mid-peripheral VFD and so-called early upper nasal step^35^. Because VFD in this region typically results from early-stage glaucoma, *SIX1/SIX6* could be associated with initial changes in GON; however, optic fissure closures—known as colobomas—also present in this area. While eyes with apparent colobomas were excluded from our analysis, subclinical cases would likely influence the statistical data. Moreover, since *PAX6* mutations are associated with coloboma formation^36^ and correlate with *SIX6* activation during eye development^37^, *SIX1/SIX6* polymorphisms could be involved in the pathophysiology of cpRNFL thinning in the inferior region. Although previous reports have not shown a connection between *SIX6* SNPs and coloboma formation^38, 39^, further genetic studies with 32-sector cpRNFLT analysis would likely lead to the identification of other genes with key roles in glaucomatous VFD development.

Our findings would suggest that we should evaluate genetic associations to cpRNFLT by dividing it into 32 sectors rather than dividing it into 4 sectors or evaluating cpRNFLT as a whole. Notably, we found a significant association between rs33912345 and inferior region cpRNFLT at 292.5–303.8° (*P* = 0.025 after Bonferroni correction) in the 32-sectored cpRNFLT analysis, whereas rs33912345 was not significantly associated with the inferior region (225–315°) in the 4-sectored analysis (*P* = 1.0 after Bonferroni correction). Furthermore, all SNPs with marginal P-values at 292.5–303.8 and 281.3–292.5° showed an equivalent contribution to cpRNFL thinning to that observed with rs33912345. In addition, rs10483727—a RNFLT-susceptible SNP not included in our analysis—showed a significant association with inferior region cpRNFLT at 281.3-303.8° (*P* = 0.016 and *P* = 0.016 after Bonferroni correction, respectively) in the 32-sector analysis, but not in the 4-sector analysis (225–315°; *P* = 1.0 after Bonferroni correction) (Supplementary Tables 1 and 2).

Based on a previous study, cpRNFL thinning at 281.3–303.8° should correspond to mid-peripheral scotoma since central VFD is associated with cpRNFL thinning at 311–40° ^27^. VFD is usually classified as central/mid-peripheral/peripheral scotoma, superior/inferior altitudinal defect, or temporal/nasal hemianopia that shared features of the clinically observed visual field patterns^40^. The altitudinal boundary is separated at 12° around optic nerve head (ONH)^41^; thus, cpRNFL should be evaluated at 12° interval or less (at least 30 sectors) to fully evaluate its correspondence to VFD patterns. To our knowledge, this is the first study evaluating the applicability of a region-based approach for GON analysis by examining the genetic contributions of glaucoma susceptibility genes with regional cpRNFLT. Therefore, genetic studies using 32-sectored cpRNFLT might reveal further associations to clinically important glaucoma phenotypes.

To date, three studies have reported significant associations of *SIX1/SIX6* polymorphisms to cpRNFL thinning in the upper and lower sectors—but not the nasal and temporal sectors—in 30 POAG cases^32^, 1,243 population controls^31^, and 231 other participants consisting of 20% normal, 44% of suspected glaucoma, and 36% confirmed glaucoma cases^30^. In contrast, our analyses used 2,306 population controls and showed that rs33912345 had the strongest association with cpRNFLT at 292.5–303.8° and a marginal association at 78.8–90.0°. RNFL thinning at 281.3–303.8° would lead to upper mid-peripheral VFDs that could lead to an early upper nasal step^27, 35^. However, further genetic studies on visual field testing are necessary to confirm whether the *SIX1/SIX6* locus would be an appropriate locus to determine the genetic factors underlying GON in the upper visual fields.

There are several limitations to this study. First, the VFD data was not obtained in our cohort. As the clinical importance of VFD has been widely accepted in GON studies, further confirmation analyses on VFD are needed. Nevertheless, we believe that cpRNFLT is an objective value with high reliability and repeatability and can facilitate the identification of hidden genetic associations for VFD. Second, only data from the right eye of subjects were analysed due to the time constraints placed on OCT acquisition; however, because both eyes are equally affected by genotype, analyses on the left eyes should yield similar findings. Third, the number of participants analysed was rather small compared to the large sample size of the Nagahama cohort. This is mainly because only subjects with genome-wide SNP data and an axial length <26 mm were included in the study. A larger sample analysis might further elucidate its associations to other regions. Fourth, population-based study methods are best evaluated with disease-free subjects. Despite the known association between *SIX1/SIX6* SNPs and glaucoma development, these patients were not specifically excluded from the study population since we did not perform visual field testing or slit lamp biomicroscopy required for this diagnosis. Thus, a subsequent study of only healthy subjects will be necessary to confirm the clinical impact of our findings. Lastly, an optimization of sector number may be beneficial in future cpRNFLT studies and it is possible that wider sectors would be sufficient to yield the same result, whereas a finer sector analysis might be able to find other associations.

In conclusion, we confirmed that rs33912345 and rs10483727—the only known cpRNFLT susceptibility SNPs—showed the strongest association with cpRNFLT of those within the *SIX1/SIX6* locus. Notably, only the 32-sector cpRNFLT analysis was capable of detecting the significant associations these SNPs with inferior cpRNFL thinning at 292.5–303.8° and 281.3–303.8°, respectively, as the results of 4-sector cpRNFLT analysis were insignificant. Collectively, this suggests that fine regional association analyses are a more effective strategy to assess glaucoma endophenotype in genomic studies and may facilitate the identification of novel genetic associations in disease pathogenesis.

## Methods

### Ethical considerations

Written informed consent was obtained from all participants. Study procedures adhered to the tenets of the Declaration of Helsinki and were approved by the ethics committee of Kyoto University Graduate School of Medicine and the Nagahama Municipal Review Board.

### Study participants

The study population consisted of healthy Japanese volunteers enrolled in the Nagahama Prospective Cohort for Comprehensive Human Bioscience (the Nagahama Study). Participants were recruited between 2008 and 2010 from the general population of Nagahama City, a rural city of 125,000 inhabitants located in central Japan. Community residents from 30–74 years of age, living independently and without physical impairment or dysfunction were eligible. Of the 9,804 included participants, nine withdrew consent to participate, and 26 were excluded because genetic analysis showed an ethnic background other than Japanese. Participants were offered a follow-up assessment 5 years after the baseline evaluation, and 8,294 of the original 9,769 cohort members participated (84.9%).

In the present study, we used a dataset of the follow-up measurement. Study subjects consisted of 2,807 individuals with genome-wide SNP genotyping, axial length, phakic status, and OCT data available by April 2016. Other exclusion criteria included prior intraocular surgery (except for cataract surgery), high myopia (axial length ≥26 mm), and presence of other ocular diseases affecting retinal nerve fibre layer thickness based on fundus photography—such as optic atrophy, anterior ischemic optic neuropathy, optic disc coloboma, retinal vein occlusion, proliferative or severe non-proliferative diabetic retinopathy, retinitis pigmentosa, and other optic neuropathies. Subjects outside of the Japanese ethnic cluster or with poor quality cpRNFL images, which could result from cataracts or small pupils, were also excluded. Glaucomatous status did not serve as exclusion criteria since visual field information was unavailable at the time of analysis. Ultimately, a total of 2,306 subjects with a ≤ 0.9 sample call rate and estimated relatedness (PI-HAT) >0.35 were included in the study population

All subjects were assessed by standardized ophthalmic examination, including an objective determination of the refractive error and corneal curvature (Autorefractor ARK-530; Nidek, Gamagori, Aichi, Japan), fundus imaging (CR-DG10; Canon, Tokyo, Japan), and axial length measurements by partial coherence interferometry (IOL Master; Carl Zeiss Meditec, Inc., Dublin, California, USA). The cpRNFL in the right eye was imaged by spectral-domain optical coherence tomography (SD-OCT) (RS-3000 advanced; Nidek, Gamagori, Aichi, Japan).

### Circumpapillary retinal nerve fibre layer thickness

The RS-3000 advanced OCT (Nidek) was used to obtain circular B-scans 11.5° in diameter (3.45 mm in the Gullstrand’s eye) centred on the optic disc, i.e., a circumpapillary scan. Each B-scan was obtained by averaging 50 images in “Regular mode” to reduce speckle noise. The cpRNFL thickness was defined as the distance between the inner border of the internal limiting membrane (ILM) and the outer border of the RNFL in B-scan images, measured automatically with built-in software, and then manually corrected for all images. We excluded eyes with extensive peripapillary atrophy (affecting cpRNFL scans), RNFL schisis, peripapillary epiretinal membrane, or thickened posterior vitreous membrane that affected segmentation, and low quality or other RNFL segmentation errors.

**Figure 1:**
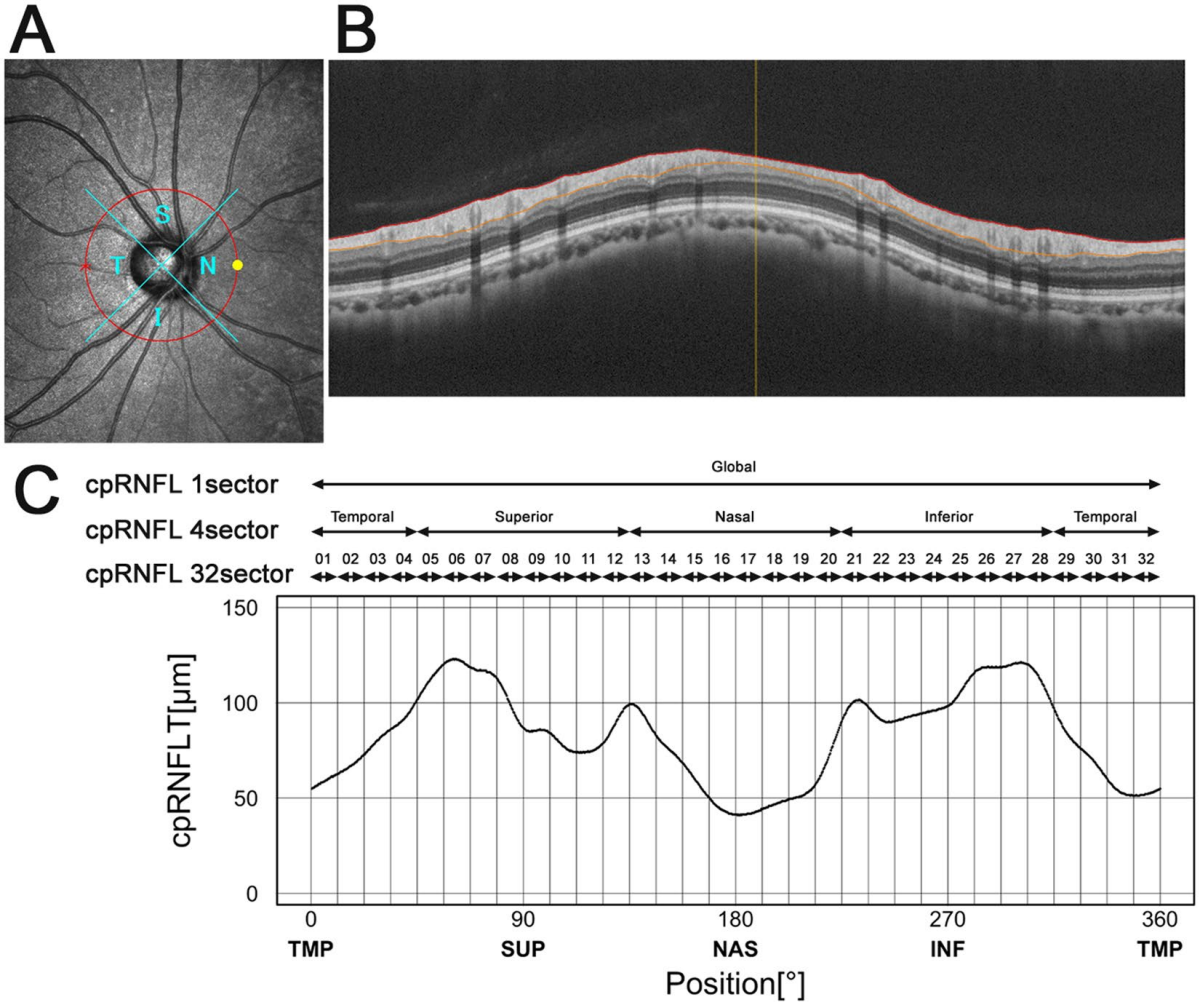
Illustration of the 32 sectors along the RS-3000 advanced (Nidek) optical coherence tomographic (OCT) circular image used for circumpapillary retinal nerve fibre layer thicknesses (cpRNFLT) analysis. (**A**,**B**) Circular B-scans 11.5° in diameter (3.45 mm in the Gullstrand’s eye) centred on the optic disc were obtained from the right eye of each participant. (**C**) cpRNFLT was measured at 1,024 points along the 360° OCT circle scan and divided into 4 or 32 sectors.

RNFL thickness was measured at 1,024 points along the 360° OCT circle scan, which were subsequently sectioned into 4 or 32 sectors (Fig. 1). Data for each sector were averaged and the associations between the *SIX1/SIX6* SNPs and mean cpRNFLT in each region were analysed.

### Genotyping and imputation

DNA samples were prepared and genotyped as described previously^42^. Briefly, 3,712 baseline samples were genotyped using at least one of the three genotyping platforms, HumanHap610K Quad Arrays, HumanOmni2.5 M Arrays, or HumanExome Arrays (Illumina, Inc., San Diego, CA). To ensure high-quality genotype data, a series of quality control (QC) filters, including sample success rate (>95%), individual call rate (>99%), minor allele frequency (MAF) cut-off (>0.01), Hardy-Weinberg equilibrium *p-*values (>1 × 10^−6^), and estimated relatedness (PI-HAT < 0.35) were applied to the data from each platform. In addition, seven ancestry outliers were identified by principal component analysis with the HapMap Phase 2 release 28 with the Japanese in Tokyo, Japan (JPT) reference dataset using EIGENSTRAT ver. 2.0. QC in PLINK^39, 43^ (ver.1.07; available at http://pngu.mgh.harvard.edu/~purcell/plink/). As a result, 3,267 baseline and 2,807 follow-up samples passed the QC filters. SNP genotype imputation was performed for the Japanese samples using MaCH^44^. Genotypes of 89 JPT samples from the 1000 Genomes Project (May 2011 release) were used as reference sequences. Imputed SNPs with an R-squared value less than 0.5 were excluded from the following association analyses.

**Figure 2:**
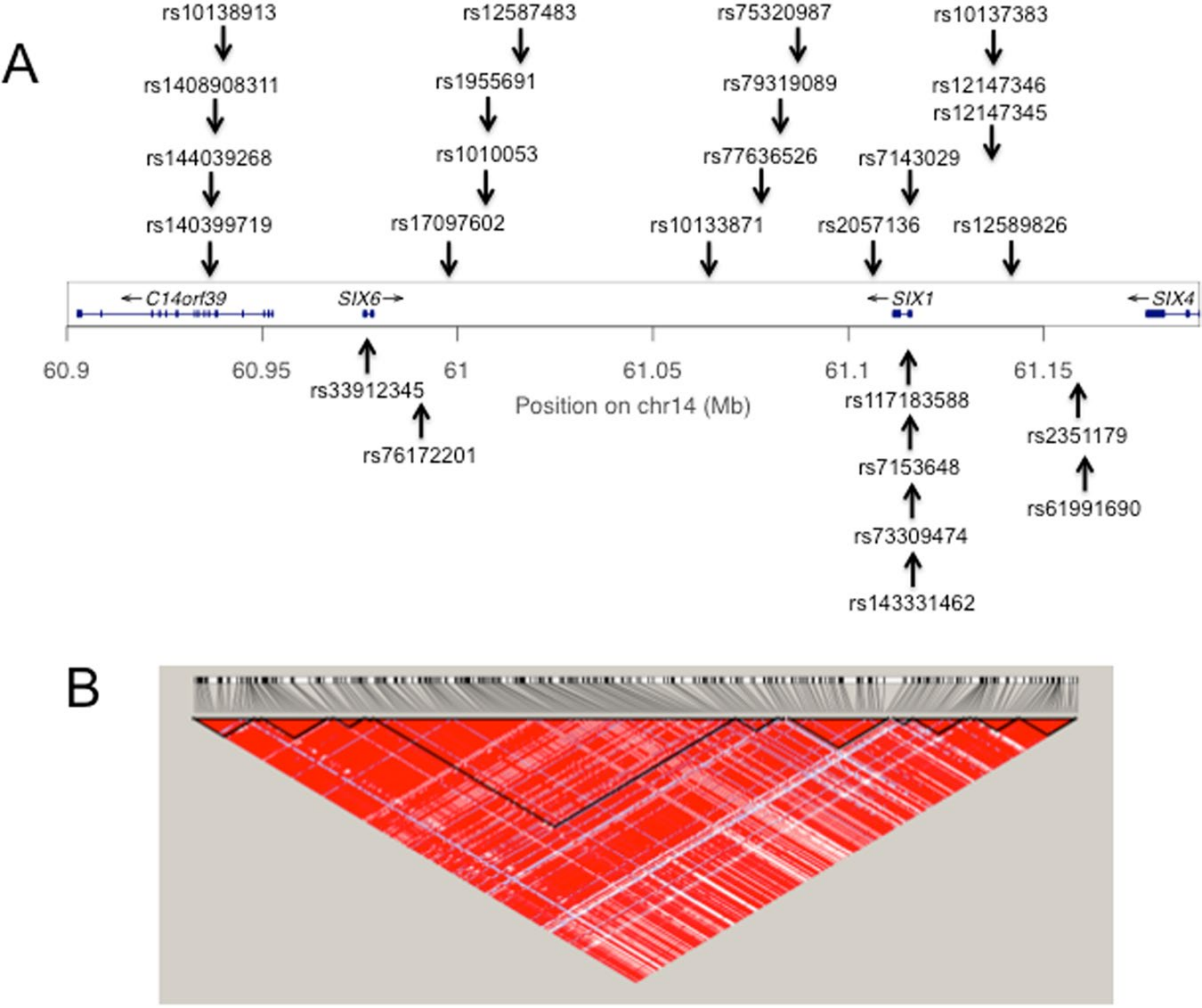
Construction of the *SIX1/SIX6* region and tagging SNPs. (**A**) Location of the 26 tagging SNPs within the *SIX1/SIX6* locus are shown relative to NCBI build 37 of the human genome. (**B**) A linkage disequilibrium map of the *SIX1/SIX6* locus ±50 kb region encompassing the 242 SNPs examined in our analysis was plotted with Haploview 4.2 software. A total of 16 haplotype blocks could be distinguished and 10 SNPs were not included in any of the blocks.

Our dataset contained 488 SNPs within ±50 kb of the *SIX1/SIX6* locus (chr14: 60925938–61166155; NCBI build 37). The Tagger program in Haploview^45^ was used to identify 26 tagging-SNPs encompassing the 242 known SNPs with an MAF > 0.05 (mean R-sq = 0.962). Since rs33912345 was previously reported as a cpRNFLT-susceptible SNP, it was selected as a tagging SNP for a positive control. The location of these 26 SNPs within the *SIX1/SIX6* locus and a linkage disequilibrium plot of this genetic region are shown in Fig. 2.

### Statistical analysis

Linear regression analyses were performed to determine the associations between regional cpRNFLT and the 26 SNP genotypes assuming additive regression models for a per-minor-allele with an adjustment for age, sex, and axial length. We evaluated regional cpRNFLT associations using 4 divided sectors and finer 32 sectors with Bonferroni corrections. P values < 6.0 × 10^−5^ (0.05/26 SNPs/32 sectors) and <4.8 × 10^−4^ (0.05/26 SNPs/4 sectors) were considered statistically significant for cpRNFL in the 32-sector and 4-sectors analyses, respectively, whereas P-values < 0.0016 (0.05/32 sectors) and <0.0125 (0.05/4 sectors) were considered marginally significant, respectively.

## Electronic supplementary material

Supplementary Tables
