# Supplementary material for: Association of SIX1/SIX6 locus polymorphisms with regional circumpapillary retinal nerve fibre layer thickness: The Nagahama study
Source: Sci Rep. 2017 Jun 29;7:4393. doi: 10.1038/s41598-017-02299-7 (PMC5491508; doi:10.1038/s41598-017-02299-7)
Supplement: Supplementary file 1 — Supplementary Tables [file 41598_2017_2299_MOESM1_ESM.pdf]

Association of *SIX1/SIX6* locus polymorphisms with regional circumpapillary retinal nerve fibre layer thickness: The Nagahama study

Munemitsu Yoshikawa[1], Kenji Yamashiro[1,2]*, Hideo Nakanishi[1], Manabu Miyata[1], Masahiro Miyake[1,3], Yoshikatsu Hosoda[1], the Nagahama Study Group[3,4,5], Yasuharu Tabara[3], Fumihiko Matsuda[3], Nagahisa Yoshimura[1]

[1]Department of Ophthalmology and Visual Sciences; [3]Center for Genomic Medicine; [4]Department of Health Informatics; [5]Department of Medical Ethics and Medical Genetics, Kyoto University Graduate School of Medicine, 54 Kawaharacho, Shogoin, Sakyo-ku, Kyoto 606-8507, Japan

[2]Department of Ophthalmology, Otsu Red Cross Hospital, Shiga, Japan

Supplementary Table 1. Associations of a *SIX1/SIX6* locus polymorphism with circumpapillary retinal nerve fibre layer thickness including 4 sectors.

CHR	SNP	BP*	Gene	Minor Allele*	Other Allele*	Global		Temporal		Superior		Nasal		Inferior	
						Beta	P†	Beta	P†	Beta	P†	Beta	P†	Beta	P†
14	rs10483727_C_T	61072875	SIX1/SIX6	C	T	-0.65	0.091	-1.10	0.013	-0.41	0.52	0.53	0.25	-1.63	0.015

CHR, chromosome; SNP, single nucleotide polymorphism; BP, base pair.
* Positions and alleles are given relative to the positive strand of NCBI build 37 of the human genome.
† Linear regression analyses were applied assuming additive effect of the per minor allele variant, adjusted for age and sex.

Supplementary Table 2. Associations of a *SIX1/SIX6* locus polymorphism and circumpapillary retinal nerve fibre layer thickness in 32 sectors.

SNP	RNFL 01	RNFL 02	RNFL 03	RNFL 04	RNFL 05	RNFL 06	RNFL 07	RNFL 08	RNFL 09	RNFL 10	RNFL 11	RNFL 12	RNFL 13	RNFL 14	RNFL 15	RNFL 16
	P*	P*	P*	P*	P*	P*	P*	P*	P*	P*	P*	P*	P*	P*	P*	P*
rs10483727	0.14	0.058	0.053	0.035	0.080	0.025	0.51	0.072	0.064	0.36	0.13	0.53	0.87	0.13	0.023	0.0085

SNP, single nucleotide polymorphism; RNFL, retinal nerve fibre layer.
RNFL01–32 starts from the temporal region at 0–11.25º and at 11.25º (=360º/32 sectors) interval (clockwise direction).
* Linear regression analyses were applied assuming additive effect of the per minor allele variant, adjusted for age and sex. Significant ($P < 6.0\times10^{-5}$) or suggestive ($P < 1.6\times10^{-3}$) associations are shown in bold.

SNP	RNFL 17	RNFL 18	RNFL 19	RNFL 20	RNFL 21	RNFL 22	RNFL 23	RNFL 24	RNFL 25	RNFL 26	RNFL 27	RNFL 28	RNFL 29	RNFL 30	RNFL 31	RNFL 32
	P*	P*	P*	P*	P*	P*	P*	P*	P*	P*	P*	P*	P*	P*	P*	P*
rs10483727	0.058	0.91	0.45	0.83	0.75	0.57	0.36	0.86	0.021	**1.9×10^{-5}**	**1.9×10^{-5}**	0.0027	0.0035	0.018	0.062	0.20
